# The İmportance of tumor markers in patients undergoing resection for lung metastasis of colorectal carcinoma

**DOI:** 10.1111/crj.13611

**Published:** 2023-04-17

**Authors:** Gizem Kececi Ozgur, Ahmet Kayahan Tekneci, Edward Hassan Bakali, Tevfik Ilker Akcam, Ayse Gul Ergonul, Ali Ozdil, Kutsal Turhan, Alpaslan Cakan, Ufuk Cagırıcı

**Affiliations:** ^1^ Department of Thoracic Surgery Ege University School of Medicine Izmir Turkey

**Keywords:** CA 19‐9 value, colorectal carcinoma, lung metastases, pulmonary metastasectomy

## Abstract

**Objectives:**

In patients with suitable conditions, complete resection is a potential curative treatment for lung metastases of colorectal cancers (CRC). Various prognostic factors affecting survival have been reported in these patients. In our study, the prognostic significance of CEA and CA19‐9 tumor markers in patients who underwent lung resection for CRC metastasis was researched.

**Methods:**

Fifty‐three patients who underwent lung resection for CRC metastasis between January 2015 and July 2021 were included in the study. The relationship between preoperative and postoperative CEA and CA19‐9 values, survival times, tumor size, and preoperative CEA and CA19‐9 levels were investigated.

**Results:**

Patients with high preoperative and postoperative CEA had shorter survival (OS) compared with patients with lower values (*p* ≤ 0.001 and *p* = 0.009, respectively). Disease‐free survival (DFS) was also shorter in patients with higher preoperative CEA values (*p* = 0.008). For patients with higher preoperative and postoperative CA 19–9 values, OS and DFS were shorter (*p* = 0.013 and *p* ≤ 0.001) and (*p* = 0.042 and *p* ≤ 0.001), respectively. There was a weak positive correlation between preoperative CEA value and tumor size (*p* = 0.008, Pearson correlation coefficient = 0.360). However, a strong positive correlation between preoperative CA19‐9 value and tumor size was discovered (*p* ≤ 0.001, Pearson correlation coefficient = 0.603).

**Conclusion:**

In our study, it was shown that preoperative‐postoperative CEA and CA19‐9 levels in patients with metastatic colon carcinoma are associated with overall survival.

## Graphical abstract

Patients with high preoperative CA19‐9 values had shorter overall and disease‐free survival times. This shows that the preoperative CA19‐9 value is a prognostic factor at least as important as CEA in lung metastasis of colorectal carcinoma.

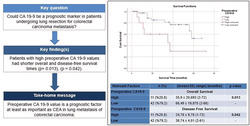

**Abbreviations**
CA19‐9: cancer antigen 19‐9
CEA: carbohydrate antigen
CRC: colorectal cancers
DFS: disease‐free survival
OS: overall survival

## 1 INTRODUCTION

Colorectal cancer (CRC) is the most common malignancy among gastrointestinal system cancers and is among the leading causes of cancer‐related deaths.^1^ Approximately 20%–25% of patients have distant metastases at the time of diagnosis.^2^, ^3^ In approximately 40%–50% of patients who underwent resection and received adjuvant treatments, metastasis may develop during the disease.^4^ Whereas the development of metastasis is accepted as the main cause of death, the most common sites of distant metastasis are the liver and lung.^5^ When left untreated, the prognosis of metastatic cases is poor, with the median survival being 5–9 months.^6^ Whereas the median survival time is 20–22 months and 5‐year survival is 5% in unresectable metastatic colon cancers with systemic spread, survival rates of longer than 5 years have been demonstrated in approximately 50% of patients with resectable pulmonary metastases.^7^ In lung metastases of colorectal cancers, complete resection is a potentially curative treatment if the primary tumor is under control, there is no evidence of extrathoracic involvement, all nodules can be removed with planned surgery, and the lung reserve is suitable for resection.^8^

It is important to determine the prognostic factors affecting survival in patients undergoing metastasectomy for CRC. Tumor stage and carcinoembryonic antigen (CEA) levels stand out as prognostic factors.^9^ However, the prognostic effect of CA 19‐9 in lung metastasis (cancer antigen) of CRC is not clear yet. For this reason, in our study, besides CEA, the prognostic significance of the CA19‐9 tumor marker and its relationship with survival was investigated in patients who underwent lung resection for CRC metastasis.

## 2 MATERIAL AND METHODS

The data of 117 patients who underwent lung resection for CRC metastasis in our clinic between January 2015 and July 2021 was analyzed retrospectively. Demographic characteristics such as age, gender, comorbidity, surgical procedures performed, preoperative and postoperative CEA, CA19‐9 levels, number and size of resected metastases, recurrence, and survival times were recorded.

Metastasectomy criteria were determined in accordance with The National Comprehensive Cancer Network (NCCN) guidelines.^8^ These criteria were: (I) Complete resection based on the anatomic location and extent of disease with the maintenance of adequate function is required, (II) the primary tumor must have been resected for cure (R0), (III) extrathoracic metastases should not be detected, but resectable extrathoracic metastases do not preclude resection. Complete (R0) resection of the tumor was performed in the patients in accordance with oncological principles. If possible, wedge resection with safe surgical margins was preferred. Because sublobar resections cause less loss of lung function compared with lobectomy,^10^ and this allows re‐applied pulmonary metastasectomy if needed in the future. If the location of the lesion was not suitable for wedge resection and the patient's respiratory functions were adequate, lobectomy was performed. In the histopathological examination of patients who underwent non‐anatomical resection, the distance from the tumor to the surgical margin was greater than 5 mm.

Patients with a diagnosis of CRC who underwent curative surgical resection for their primary tumor, with isolated lung metastases or with resectable extrathoracic metastases, whose location of lung metastases could be resected with adequate surgical margins (wedge resection or anatomical resections), and whose pulmonary function tests were suitable for lung resection were included in the study. Patients were excluded if extrathoracic metastases were detected radiologically and these metastases were not resectable. Patients who had undergone enucleation were excluded because of the location of the lesion, and pulmonary function tests were not suitable for a wider resection. This was because, unlike wedge resection and anatomical resections, the enucleation method could not provide safe surgical margins. In addition, patients whose tumor marker values could not be evaluated in the first month postoperatively because they did not attend routine controls were also excluded. Of the 117 patients who had undergone pulmonary metastasectomy for CRC, data from 53 patients who met the inclusion and exclusion criteria were analyzed.

Fasting venous blood samples (2 mL) were collected from all patients on preoperative the day before the operation and postoperative first month. Tumor markers were measured using the electrochemiluminescence immunoassay method. Serum CEA (carcinoembryonic antigen electrochemiluminescence immunoassay “ECLIA”) and serum CA 19‐9 (carbohydrate electrochemiluminescence immunoassay “ECLIA”) were all measured in the same laboratory. All lab tests were performed in accordance with the standard operating procedures. Analyzes were performed on the day of sample collection, and tumor marker levels were recorded. Cut‐off values were accepted as 5 μg/L for CEA and 27 U/mL for CA19‐9.

In the first step of the study, the change in preoperative CEA and CA 19‐9 values in the first‐month check‐up after metastasectomy was statistically analyzed.

In the second step of the study, patients were divided into two groups: those with a preoperative CEA value below and above 5 μg/L, as (Group_pre‐CEA≤_) (Group_pre‐CEA>_), respectively. Likewise, those with a postoperative CEA value below and above 5 μg/L, as (Group_post‐CEA≤_) (Group_post‐CEA>_), respectively. The relationship between preoperative and postoperative CEA values and survival times was examined.

Patients with preoperative CA19‐9 values below and above 27 U/mL were categorized as (Group_pre‐CA‐19‐9≤_) and (Group_pre‐CA‐19‐9>_), respectively. Likewise, patients with postoperative CA19‐9 values below and above 27 U/mL were categorized as (Group_post‐CA‐19‐9≤_) and (Group_post‐CA‐19‐9>_), respectively. The relationship between preoperative and postoperative CA19‐9 values and survival times was examined.

In the third and last step of the study, the correlation between the tumor sizes of the patients and the preoperative tumor markers, such as CEA and CA 19–9, was investigated.

### 2.1 Statistical analyses

Data were analyzed using the SPSS 26.0 (IBM statistics for Windows version 26, IBM Corporation, Armonk, New York, United States) and PAST (Hammer, Ø., Harper, D.A.T., Ryan, P.D. 2001. Paleontological Statistics) software packages. Quantitative data were expressed in mean ± SD (standard deviation) and the median range (maximum‐minimum) values. Categorical data were expressed in n (number) and percentage (%). The dependent sample T‐test and Wilcoxon test were used to compare preoperative and postoperative dependent quantitative parameters. The chi‐square test, Student's t‐test, and Mann–Whitney U test were used to evaluate the statistical differences between the groups. Survival analyses were performed using the Kaplan–Meier method and Loran (Mantel–Cox) test. Data were evaluated with a 95% confidence interval, and statistical significance was based on a value of *p* < 0.05. Cox regression analyses were performed, and hazard ratio values were determined for possible variables. *P* value <0.05 was considered significant.

## 3 RESULTS

Whereas 40 (75.5%) of the patients were 60 years or older, the mean age of all patients was 64.60 ± 9.33 (39–83) years. Whereas 18 of the patients (34%) had a smoking history of more than five pack years, all of the patients who smoked were ex‐smokers. When the metastatic lesions in the lung were examined according to their localization, it was determined that lesions were located in the right lung in 34 (64.1%) patients, in the left lung in 17 (32.1%) patients, and in the bilateral lungs in two (3.8%) patients. In 17 (32.1%) patients, it was determined that most of the lesions were located in the lower lobe of the right lung.

Sublobar resection was performed in 42 (79.3%) patients, and anatomical resection was performed in 11 (21.7%) patients. Postoperative complications developed in six of 53 patients (%11.3). Of these, four were prolonged air drainage (longer than 7 days), and two were arrhythmias. Whereas one metastasis was detected in 41 (77.4) cases, two metastatic foci were observed in six (11.3%) and three or more in six of them. Whereas recurrence or metastasis developed in 19 (35.8%) of the cases after the operation, 13 (68.4%) of these lesions were in the lung, four (21.1%) in the liver, and two (10.5%) were located in the colon. Whereas metastatic lesions were observed to be 2 cm or more in 20 (37.7%) patients, the mean tumor size of all patients was determined to be 18.13 ± 12.74 (1–70) mm (Table 1). Mortality developed in 15 (28.3%) of the patients during the follow‐up, and the overall survival (OS) of the patients was found to be 52.38 ± 20.06 (2–72) months.

**TABLE 1:** Demographic characteristics of patients.

Variables	Number of patients (*n* = 53)	Rate (%)
Gender		
Male	29	54.7
Female	24	45.3
Age		
Under 60	13	24.5
Over 60	40	75.5
Average age (mean ± SD, range) (year)	64.60 ± 9.332 (39–83)	
Localization of metastatic lesions		
Right lung	32	39.5
Left lung	17	20.9
Bilateral lungs	16	19.7
Number of metastases		
One	41	77.4
Two	6	11.3
Three or more	6	11.3
Tumor size		
<20 mm	13	24.5
≥20 mm	40	75.5
Average size (mean ± SD, range) (mm)	18.13 ± 12.748 (1–70) mm	
Surgical procedure		
Anatomic resection	11	21.7
Sublobar resection	42	79.3
Relapse or metastasis		
Present	19	35.8
Absent	34	64.2
Localization of relapse or metastasis		
Lung	13	68.4
Liver	4	21.1
Colon	2	10.5
Mortality		
Present	15	28.3
Absent	38	71.7
Preoperative CEA		
High	16	30.2
Low	37	69.8
(mean ± SD, range) (μg/L)	10.078 ± 20.848 (1.2–107.4) μg/L	
Postoperative CEA		
High	14	26.4
Low	39	73.6
(mean ± SD, range) (μg/L)	9.041 ± 27.744 (1.1–197) μg/L	
Preoperative CA19‐9		
High	11	20.8
Low	42	79.2
(mean ± SD, range) (U/mL)	18.884 ± 18.108 (0.5–99) U/mL	
Postoperative CA19‐9		
High	9	17
Low	44	83
(mean ± SD, range) (U/mL)	17.095 ± 15.755 (0.5–92) U/mL	

Abbreviations: CA19‐9, carbohydrate antigen 19‐9; CEA, carcinoembryonic antigen.

The mean preoperative CEA values of the patients followed up with tumor markers in the preoperative and postoperative first month were 10.07 ± 20.84 (1.22–107.4) μg/L, and 16 (30.2%) cases were above the 5 μg/L reference value. In the first month postoperatively, the mean CEA values were found to be 9.04 ± 27.74 (1.10–197) μg/L, and it was above the reference value in 14 (26.4%) cases (Table 1). Mean preoperative CA19‐9 values were found to be 18.88 ± 18.10 (0.5–99) U/mL and were above the reference value of 27 U/mL in 11 (20.8%) cases. In the first month postoperatively, the mean of CA19‐9 values was found to be 17.09 ± 15.75 (0.5–92) U/mL, and it was above the reference value in 9 (17%) cases (Table 1). Although a decrease was observed in the mean values of both tumor markers in the postoperative period, this decrease was not statistically significant.

The mean preoperative CEA value of 15 (28.3%) patients who developed mortality was 18.88 ± 32.29 (1.66 to 107.40) μg/L, whereas the mean value of those who survived was 6.06 ± 13.12 (1.22–73.60) μg/L. The mean postoperative CEA value in patients who developed mortality was 10.02 ± 15.21 (1.39–60.86) μg/L, whereas the mean value in survivors was 8.65 ± 31.52 (1.10–197) μg/L. The mean preoperative CA19‐9 value was 29.41 ± 26.03 (0.5–99) U/mL in patients who developed mortality, whereas the mean value in survivors was 14.72 ± 11.90 (0.5–56.11) U/mL. The mean postoperative CA19‐9 value was 23.97 ± 15.54 (1.81–49) U/mL in those who developed mortality and 14.37 ± 15.18 (0.50–92) U/mL in those who survived. Preoperative CEA, postoperative CEA, preoperative CA19‐9, and postoperative CA19‐9 values were higher in patients who developed mortality compared with those who survived (*p* = 0.004, *p* = 0.035, *p* = 0.034, *p* = 0.015). In addition, the mean preoperative CA19‐9 value of 19 (35.8%) patients with a tumor size of 2 cm or more was 29.00 ± 23.51 (2.80–99) U/mL, whereas the mean value of those with a tumor size less than 2 cm was 13.22 ± 11.09 (0.5–48) μg/L. Likewise, the postoperative mean CA19‐9 value was 20.64 ± 12.27 (4.90–48.87) U/mL in patients with large tumor size, whereas it was 15.11 ± 17.25 (0.50–92) U/mL in survivors. Preoperative CA19‐9 and postoperative CA19‐9 values were higher in patients with tumor size of 2 cm and above than in patients with small tumors (*p* = 0.002, *p* = 0.028). When the patients were grouped according to gender, number of metastases, recurrence status, and smoking, no significant difference was observed between the groups in terms of preoperative and postoperative tumor markers.

Recurrence or metastasis developed in seven (43.8%) of 16 patients with high preoperative CEA and in 12 (32.4%) of 37 patients with low preoperative CEA levels. Recurrence or metastasis developed in four (28.6%) of 14 patients with high postoperative CEA and in 15 (38.5%) of 39 patients with low postoperative CEA levels. Although a difference between the groups was observed, it was not statistically significant. Recurrence or metastasis developed in five (43.5%) of 11 patients with a high preoperative CA19‐9 value and in 14 (33.3%) of 42 patients with a low CA19‐9 value. Recurrence or metastasis developed in four (44.4%) of 9 patients with a high postoperative CA19‐9 value and in 15 (34.1%) of 44 patients with a low CA19‐9 value. The difference between the groups, though noted, was not statistically significant.

The general survival of patients with high preoperative CEA value was 26,57 ± 5.18 (3–58) months, and in those with low CEA value, this period was 62.75 ± 3.73 (2–72) months, and this difference between the two groups was statistically significant (*p* ≤ 0.001). The general survival of patients with high postoperative CEA value was 31.68 ± 7.03 (3–61) months, and for those with low CEA value was 57.51 ± 4.10 (2–72) months, and this difference between the two groups was statistically significant (p = 0.009) (Table 2). In patients with high preoperative CEA value, disease‐free survival (DFS) was 22.16 ± 7.25 (2–58) months, whereas this period was 45.67 ± 5.21 (1–72) months in cases with low CEA value. This difference between the two groups was statistically significant (*p* = 0.008). The DFS of patients with high postoperative CEA value was 30.80 ± 7.68 (3–61) months and 40.34 ± 5.04 (1–72) months in those cases with low CEA value. The difference between the two groups was not statistically significant (*p* = 0.455) (Table 3).

**TABLE 2:** Overall survival (OS) analyses.

	*n* (%)	Survival (mean ± SD, range) (months)	*P*‐values
Preoperative CEA			
High	16 (30.2%)	26.57 ± 5.18 (3–58)	≤0.001
Low	37 (69.8%)	62.75 ± 3.73 (2–72)	‐
Postoperative CEA			
High	14 (26.4%)	31.68 ± 7.03 (3–61)	0.009
Low	39 (73.6%)	57.51 ± 4.10 (2–72)	‐
Preoperative CA19‐9			
High	11 (20.8%)	35.90 ± 8.87 (2–72)	0.013
Low	42 (79.2%)	68.49 ± 3.71 (2–68)	‐
Postoperative CA19‐9			
High	9 (17%)	19.88 ± 5.16 (5–37)	≤0.001
Low	44 (83%)	59.35 ± 3.75 (2–72)	‐
Smoking history			
Yes	18 (34%)	26.30 ± 4.20 (5–72)	0.013
No	35 (66%)	56.92 ± 3.89 (2–37)	‐

Abbreviations: CA19‐9, carbohydrate antigen 19‐9; CEA, carcinoembryonic antigen.

**TABLE 3:** Diseases free survival analyses.

	*n* (%)	Survival (mean ± SD, range) (months)	*P*‐values
Preoperative CEA			
High	16 (30.2%)	22.16 ± 7.25 (2–58)	0.008
Low	37 (69.8%)	45.67 ± 5.21 (1–72)	‐
Postoperative CEA			
High	14 (26.4%)	30.80 ± 7.68 (3–61)	0.455
Low	39 (73.6%)	40.34 ± 5.04 (1–72)	‐
Preoperative CA19‐9			
High	11 (20.8%)	24.78 ± 9.78 (1–72)	0.042
Low	42 (79.2%)	38.74 ± 4.01 (2–61)	‐
Postoperative CA19‐9			
High	9 (17%)	8.44 ± 2.26 (1–17)	≤0.001
Low	44 (83%)	44.12 ± 4.80 (2–72)	‐

Abbreviations: CA19‐9, carbohydrate antigen 19‐9; CEA, carcinoembryonic antigen.

The general survival of patients with high preoperative CA19‐9 value was 35.90 ± 8.87 (2–72) months, and in cases with low CA19‐9 was 54.58 ± 3.71 (2–68) months. The difference between the two groups was statistically significant (*p* = 0.013). The general survival of patients with high postoperative CA19‐9 value was 19.88 ± 5.16 (5–37) months, and in cases with low CA19‐9 was 59.35 ± 3.75 (2–72) months. The difference between the two groups was statistically significant (*p* ≤ 0.001) (Table 2). The DFS of patients with high and low preoperative CA19‐9 values was 24.78 ± 9.78 (1–72) and 38.74 ± 4.01 (2–61) months, respectively. The difference between the two groups was statistically significant (*p* = 0.042). The DFS of patients with high postoperative CA19‐9 value was 8.44 ± 2.26 (1–17) months, whereas in cases with low CA19‐9 value was 44.12 ± 4.80 (2–72) months. The difference between the two groups was statistically significant (*p* ≤ 0.001) (Table 3).

In addition, OS was 26.30 ± 4.20 (5–72) months in patients with a history of smoking and 56.92 ± 3.89 (2–37) months in non‐smokers. The difference between the two groups was statistically significant (*p* = 0.013) (Table 2).

**FIGURE 1:**
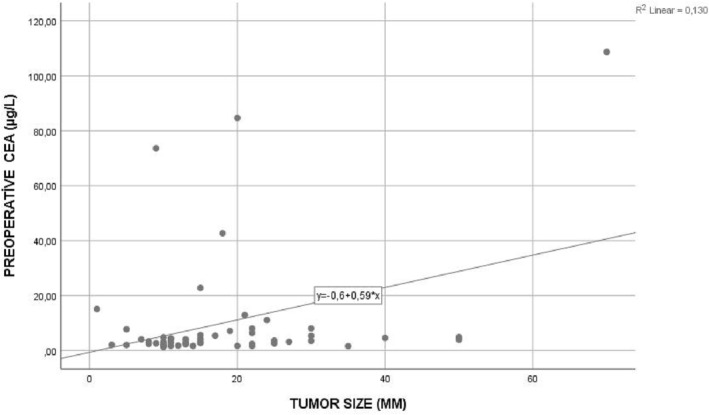
Correlation relationship between preoperative carcinoembryonic antigen (CEA) value and tumor size.

**FIGURE 2:**
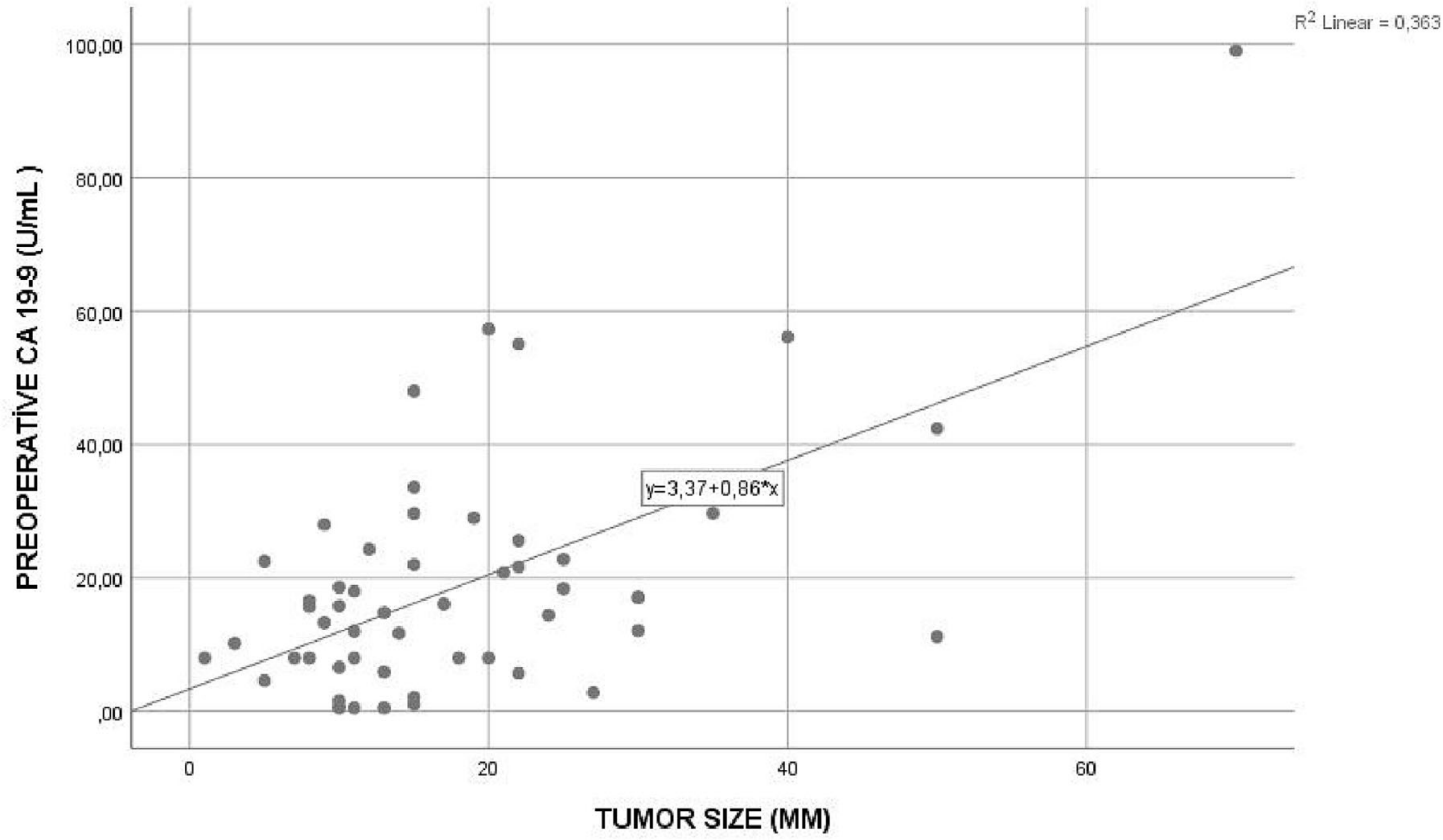
Correlation relationship between preoperative carbohydrate antigen 19‐9 (CA19‐9) value and tumor size.

The correlation relationship between the tumor dimensions of the patients and the preoperative tumor markers was investigated. Preoperative tumor markers and large metastatic lesions were found to have a positive correlation. Whereas a weak correlation was found between preoperative CEA value and tumor size (*p* = 0.008, Pearson correlation coefficient = 0.360) (Figure 1), preoperative CA19‐9 value was found to have a strong positive correlation with tumor size (P ≤ 0.001, Pearson correlation coefficient = 0.603) (Figure 2). It has therefore been established that as the tumor size increased, preoperative CEA and CA19‐9 values increased proportionally.

A lot of multi‐module analyses have been performed with the factors considered associated with patients' survival time, such as the number of metastases, recurrence, preoperative and postoperative CEA, and CA19‐9 values. As a result of the analysis, the risk of death of CRC patients who had lung resection due to metastasis is 29.17 times in patients with high CA 19‐9 in the postoperative process and 5.4 times higher in patients with high preoperative CEA value. In patients with two or more metastases, the risk of death was found to be 6.5 times, and in those with a tumor size of 2 cm or more was found to be 2.1 times higher (Table 4).

**TABLE 4:** Evaluation of factors affecting survival by Cox regression analysis.

Patient groups	Hazard ratio	%95 CL Lower–upper	*P*‐values
Postoperative CA19‐9 ≥ 27 U/mL	29.7	2.8–294.9	0.004
Preoperative CEA ≥ 5 μg/L	5.4	1.2–22.9	0.021
Tumor size ≥20 mm	2.1	0.5–8.1	0.023
Metastasis count ≥2	6.5	1.3–32.6	0.022

Abbreviations: CA19‐9, carbohydrate antigen 19‐9; CEA, carcinoembryonic antigen.

## 4 DISCUSSION

Pulmonary metastasectomy has become a commonly accepted treatment strategy for many CRC patients with metastases limited to the lung. Sakamaki et al.^11^ reported in their studies in patients with pulmonary metastasectomy due to lung metastasis of CRC that the average survival was 48 months. In our study, concurring with the literature findings, the average survival was 52 months. In the literature, various factors affecting survival and prognosis in lung metastases due to colorectal cancer have been reported. In these parameters, age, gender, localization, stage of primary disease, level of primary disease, size of metastatic nodules, number of metastatic nodules, lymph gland metastasis evaluation, recurrent metastases, preoperative CEA level, neoadjuvant chemotherapy, type and number of neoadjuvant chemotherapy sessions, and type and number of adjuvant chemotherapy sessions received after surgical resection constitute the main parameters of life expectancy. Gonzalez et al.,^12^ in a meta‐analysis conducted to evaluate the risk factors affecting survival in patients with lung metastases of colorectal carcinoma, revealed four parameters as important predictors of poor outcomes after lung metastasectomy. These synchronous lung metastases, involvement of thoracic lymph nodes, presence of multiple lung metastases and high preoperative CEA level. A highly reliable biomarker in the screening, diagnosis, follow‐up, and prognosis of colorectal carcinomas has not yet been determined. However, the search for markers provides great benefits for the clinician and the patient, despite varying sensitivity and specificity rates. In particular, the combined use of some markers has been shown to provide significant benefits in these cases.

CEA is a high molecular weight glycoprotein and is the most widely used tumor marker in colorectal cancer patients.^13^ Nowadays, CEA level is considered as important as tumor (T), lymph node (N), and metastasis (M) (TNM) stage.^14^, ^15^, ^16^, ^17^, ^18^, ^19^ However, even in colorectal carcinoma patients with normal CEA levels, the recurrence rate is more than 20%.^20^ Increased CEA concentrations are rarely observed in the early stages of the disease but, if detected, may indicate a negative prognosis.^21^ In the literature, it has been shown that an elevated CEA level before lung metastasectomy is associated with a worse prognosis in patients with colorectal cancer and lung metastases.^22^, ^23^, ^24^, ^25^ In our study, similar to the literature, it was determined that the survival time was shorter in the patient group with high preoperative CEA than in those with low preoperative CEA values (*p* ≤ 0.001). Similar to general survival, disease‐free survival times were found to be shorter in the patient group with a high preoperative CEA value (*p* = 0.008). Whereas the OS time was shorter in cases with high postoperative CEA (*p* = 0.009), although there was a difference in terms of disease‐free survival, it was not statistically significant (*p* = 0.455).

CA19‐9 is a circulating antigen that functions as an adhesion molecule and plays a role in tumor progression. It is not routinely used in CRC patients because it is less sensitive than the CEA test.^18^ However, several studies have suggested that analyzing CA19‐9 and CEA together may increase prognostic sensitivity.^26^, ^27^ Lin et al.^28^ stated that in patients with preoperative high CA19‐9 value in lung metastasis of CRC, disease‐free survival was shorter, and recurrence was higher in this group. In our study, recurrence was detected in 45.5% of patients with a high preoperative CA19‐9 value. It was observed that the survival time was shorter in the patient group with high preoperative CA19‐9 value than those with low preoperative CA19‐9 (*p* = 0.013). Similar to general survival, the disease‐free survival times were shorter in the patient group with a high preoperative CA19‐9 value (*p* = 0.042). OS and disease‐free survival were also shorter in cases with postoperative elevated CA19‐9 (*p* ≤ 0.001). As a result of multi‐module analyses done in CRC patients who underwent resection for lung metastasis, the risk of death was 29 times higher in patients with high postoperative CA19‐9, whereas in patients with a high preoperative CEA value, it was 5.4 times higher.

In addition, preoperative CA19‐9 and postoperative CA19‐9 values were higher in patients with a tumor size of 2 cm and above than in patients with small tumors (*p* = 0.002, *p* = 0.028). Also, it was determined that there was a weak positive correlation between preoperative CEA value and tumor size (*p* = 0.008, Pearson correlation coefficient = 0.360) (Figure 1), and a strong positive correlation between preoperative CA19–9 levels and tumor size (*p* ≤ 0.001, Pearson correlation coefficient = 0.603 (Figure 2).

## 5 CONCLUSION

The prognostic importance of CEA values in primary CRC is known. There are also studies on its prognostic importance in CRC with lung metastasis. In our study, the prognostic importance of preoperative and postoperative CEA value was emphasized, again in line with the literature. The prognostic significance of CA19‐9 has not been clearly defined, and there are not enough studies in the literature on this subject. The results of our study showed that CA19‐9 could be a prognostic factor at least as good as the CEA value. In addition to this, tumor size and CA19‐9 show a stronger correlation. Data from this study suggest that the CA19‐9 value is an important prognostic factor in CRC patients with lung metastases whose primary tumor site is under control.

## 6 LIMITATIONS

There are some limitations of this study that should be kept in view when interpreting. Firstly, this is a retrospective and single‐center study; therefore, the methodology used cannot be generalized to other centers. Secondly, the number of cases in the study was small but sufficient for statistical evaluation.

## AUTHOR CONTRIBUTIONS

**Gizem Kececi Ozgur:** Designed the research; conceptualization; data curation; formal analysis; investigation; methodology; supervision; validation; visualization; writing—original draft; writing—review & editing; writing—original draft. **Ahmet Kayahan Tekneci:** Designed the research; conceptualization; data curation; formal analysis; investigation; methodology; validation; visualization; writing—review & editing; writing—original draft. **Hassan Edwars Bakali:** data curation; formal analysis; investigation; methodology; validation; visualization; writing—review & editing; writing—original draft. **Tevfik Ilker Akcam:** designed the research; conceptualization; data curation; formal analysis; investigation; methodology; supervision; validation; visualization; writing—original draft; writing—review & editing; writing—original draft. **Ayse Gul Ergonul:** Investigation; supervision; writing—review & editing; writing—original draft. **Ali Ozdil:** Investigation; supervision; writing—review & editing; writing—original draft. **Kutsal Turhan:** Investigation; supervision; writing—review & editing; writing—original draft. **Alpaslan Cakan:** Investigation; supervision; writing—review & editing; writing—original draft. **Ufuk Cagirici:** Investigation; supervision; writing—review & editing; writing—original draft.

## CONFLICT OF INTEREST STATEMENT

The authors declare no conflict of interest with respect to the authorship and/or publication of this article.

## ETHICS STATEMENT

Written informed consent was obtained from each patient. The study was conducted in accordance with the principles of the Declaration of Helsinki. Approval was obtained from the local Ethics Committee for the study, which was designed as a retrospective study, no. 22‐5T/18. Ege University Faculty of Medicine Research Ethics Committees https://aek-med.ege.edu.tr/.

## Data Availability

The dataset of the research presented in this article is under record. The corresponding authors can be contacted to reach the required data network.

## References

[1] Sung H , Ferlay J , Siegel RL , et al. Global cancer statistics 2020: GLOBOCAN estimates of incidence and mortality worldwide for 36 cancers in 185 countries. CA Cancer J Clin. 2021;71(3):209‐249. doi:10.3322/caac.21660 33538338

[2] Kemeny N , Fata F . Arterial, portal, or systemic chemotherapy for patients with hepatic metastasis of colorectal carcinoma. J Hepatobiliary Pancreat Surg. 1999;6(1):39‐49. doi:10.1007/s005340050082 10436236

[3] Van der Geest LG , Lam‐Boer J , Koopman M , Verhoef C , Elferink MA , de Wilt JH . Nationwide trends in incidence, treatment and survival of colorectal cancer patients with synchronous metastases. Clin Exp Metastasis. 2015;32(5):457‐465. doi:10.1007/s10585-015-9719-0 25899064

[4] Stangl R , Altendorf‐Hofmann A , Charnley RM , Scheele J . Factors influencing the natural history of colorectal liver metastases. Lancet. 1994;343(8910):1405‐1410. doi:10.1016/S0140-6736(94)92529-1 7515134

[5] Riihimäki M , Hemminki A , Sundquist J , Hemminki K . Patterns of metastasis in colon and rectal cancer. Sci Rep. 2016;6(1):29765. doi:10.1038/srep29765 27416752 PMC4945942

[6] Günel N , Yamaç D , Akçalı Z , Taneri F , Ouz M . The clinico‐pathologic characteristics of colorectal cancer under 50 years of age; experience of an oncology center. Tumori. 2001;87(2):74‐77. doi:10.1177/030089160108700202 11401210

[7] Inoue M , Ohta M , Iuchi K , et al. Thoracic surgery study Group of Osaka University. Benefits of surgery for patients with pulmonary metastases from colorectal carcinoma. Ann Thorac Surg. 2004;78(1):238‐244. doi:10.1016/j.athoracsur.2004.02.017 15223436

[8] National Comprehensive Cancer Network . NCCN guidelines for colon cancer, version 2.2022. https://www.nccn.org/professionals/physician_gls/pdf/colon.pdf

[9] Compton CC , Fielding LP , Burgart LJ , et al. Prognostic factors in colorectal cancer. College of American Pathologists Consensus Statement 1999. Arch Pathol Lab Med. 2000;124(7):979‐994. doi:10.5858/2000-124-0979-PFICC 10888773

[10] Pezzuto A , Trabalza Marinucci B , Ricci A , et al. Predictors of respiratory failure after thoracic surgery: a retrospective cohort study with comparison between lobar and sub‐lobar resection. J Int Med Res. 2022;50(6):3000605221094531. doi:10.1177/03000605221094531 35768901 PMC9251996

[11] Sakamaki Y , Ishida D , Tanaka R . Prognosis of patients with recurrence after pulmonary metastasectomy for colorectal cancer. Gen Thorac Cardiovasc Surg. 2020;68(10):1172‐1178. doi:10.1007/s11748-020-01368-5 32323124

[12] Gonzalez M , Poncet A , Combescure C , Robert J , Ris HB , Gervaz P . Risk factors for survival after lung metastasectomy in colorectal cancer patients: a systematic review and meta‐analysis. Ann Surg Oncol. 2013;20(2):572‐579. doi:10.1245/s10434-012-2726-3 23104709

[13] McKeown E , Nelson DW , Johnson EK , et al. Current approaches and challenges for monitoring treatment response in colon and rectal cancer. J Cancer. 2014;5(1):31‐43. doi:10.7150/jca.7987 24396496 PMC3881219

[14] Desch CE , Benson AB 3rd , Somerfield MR , et al. American Society of Clinical Oncology. Colorectal cancer surveillance: 2005 update of an American Society of Clinical Oncology practice guideline. J Clin Oncol. 2005;23(33):8512‐8519. doi:10.1200/JCO.2005.04.0063 16260687

[15] Compton C , Fenoglio‐Preiser CM , Pettigrew N , Fielding LP . American joint committee on cancer prognostic factors consensus conference: colorectal working group. Cancer. 2000;88(7):1739‐1757. doi:10.1002/(SICI)1097-0142(20000401)88:7003C1739::AID-CNCR30003E3.0.CO;2-T 10738234

[16] Bast RC Jr , Ravdin P , Hayes DF , et al. American Society of Clinical Oncology tumor markers expert panel. 2000 update of recommendations for the use of tumor markers in breast and colorectal cancer: clinical practice guidelines of the American Society of Clinical Oncology. J Clin Oncol. 2001;19(6):1865‐1878. doi:10.1200/JCO.2001.19.6.1865 11251019

[17] Duffy MJ , van Dalen A , Haglund C , et al. Clinical utility of biochemical markers in colorectal cancer: European group on tumour markers (EGTM) guidelines. Eur J Cancer. 2003;39(6):718‐727. doi:10.1016/S0959-8049(02)00811-0 12651195

[18] Locker GY , Hamilton S , Harris J , et al. ASCO 2006 update of recommendations for the use of tumor markers in gastrointestinal cancer. J Clin Oncol. 2006;24(33):5313‐5327. doi:10.1200/JCO.2006.08.2644 17060676

[19] Duffy MJ , van Dalen A , Haglund C , et al. Tumour markers in colorectal cancer: European group on tumour markers (EGTM) guidelines for clinical use. Eur J Cancer. 2007;43(9):1348‐1360. doi:10.1016/j.ejca.2007.03.021 17512720

[20] Lin JK , Lin CC , Yang SH , et al. Early postoperative CEA level is a better prognostic indicator than is preoperative CEA level in predicting prognosis of patients with curable colorectal cancer. Int J Colorectal Dis. 2011;26(9):1135‐1141. doi:10.1007/s00384-011-1209-5 21538056

[21] Kahi CJ , Anderson JC , Rex DK . Screening and surveillance for colorectal cancer: state of the art. Gastrointest Endosc. 2013;77(3):335‐350. doi:10.1016/j.gie.2013.01.002 23410695

[22] Sakamoto T , Tsubota N , Iwanaga K , Yuki T , Matsuoka H , Yoshimura M . Pulmonary resection for metastases from colorectal cancer. Chest. 2001;119(4):1069‐1072. doi:10.1378/chest.119.4.1069 11296171

[23] Saito Y , Omiya H , Kohno K , et al. Pulmonary metastasectomy for 165 patients with colorectal carcinoma: a prognostic assessment. J Thorac Cardiovasc Surg. 2002;124(5):1007‐1013. doi:10.1067/mtc.2002.125165 12407386

[24] Lee WS , Yun SH , Chun HK , et al. Pulmonary resection for metastases from colorectal cancer: prognostic factors and survival. Int J Colorectal Dis. 2007;22(6):699‐704. doi:10.1007/s00384-006-0218-2 17109105

[25] Headrick JR , Miller DL , Nagorney DM , et al. Surgical treatment of hepatic and pulmonary metastases from colon cancer. Ann Thorac Surg. 2001;71(3):975‐980. doi:10.1016/S0003-4975(00)02522-4 11269484

[26] Chen CC , Yang SH , Lin JK , et al. Is it reasonable to add preoperative serum level of CEA and CA19‐9 to staging for colorectal cancer? J Surg Res. 2005;124(2):169‐174. doi:10.1016/j.jss.2004.08.013 15820244

[27] Nozoe T , Rikimaru T , Mori E , Okuyama T , Takahashi I . Increase in both CEA and CA19‐9 in sera is an independent prognostic indicator in colorectal carcinoma. J Surg Oncol. 2006;94(2):132‐137. doi:10.1002/jso.20577 16847905

[28] Lin PC , Lin JK , Lin CC , et al. Carbohydrate antigen 19‐9 is a valuable prognostic factor in colorectal cancer patients with normal levels of carcinoembryonic antigen and may help predict lung metastasis. Int J Colorectal Dis. 2012;27(10):1333‐1338. doi:10.1007/s00384-012-1447-1 22426691

